# Supplementary material for: A genome-wide screen in macrophages identifies PTEN as required for myeloid restriction of Listeria monocytogenes infection
Source: PLoS Pathog. 2023 May 22;19(5):e1011058. doi: 10.1371/journal.ppat.1011058 (PMC10237667; doi:10.1371/journal.ppat.1011058)
Supplement: S2 Table — (DOCX) [file ppat.1011058.s002.docx]

**S2 Table. Bacterial strains used in this study**

|  | **Species** | **Strain** | **Description** | **Reference or source** |
| --- | --- | --- | --- | --- |
| MLR-E0848 | *E. coli* | SM10 | pPL1.pHyper-GFP | This work |
| MLR-E0868 | *E. coli* | SM10 | pPL2t.pHyper-RFP | This work |
| MLR-E1095 | *E. coli* | SM10 | pPL2t.p*actA*-RFP | This work |
| MLR-E1096 | *E. coli* | XL1 | lentiCRISPR.*Taf4a*-sgRNA1 | This work |
| MLR-E1097 | *E. coli* | XL1 | lentiCRISPR.*Taf4a*-sgRNA2 | This work |
| MLR-E1098 | *E. coli* | XL1 | lentiCRISPR.*Supt20*-sgRNA1 | This work |
| MLR-E1099 | *E. coli* | XL1 | lentiCRISPR.*Supt20*-sgRNA2 | This work |
| MLR-E1100 | *E. coli* | XL1 | lentiCRISPR.*Rfwd2*-sgRNA1 | This work |
| MLR-E1101 | *E. coli* | XL1 | lentiCRISPR.*Rfwd2*-sgRNA2 | This work |
| MLR-E1102 | *E. coli* | XL1 | lentiCRISPR.*Dyrk1A*-sgRNA1 | This work |
| MLR-E1103 | *E. coli* | XL1 | lentiCRISPR.*Dyrk1a*-sgRNA2 | This work |
| MLR-E1104 | *E. coli* | XL1 | lentiCRISPR.*Rragc*-sgRNA1 | This work |
| MLR-E1105 | *E. coli* | XL1 | lentiCRISPR.*Rragc*-sgRNA2 | This work |
| MLR-E1106 | *E. coli* | XL1 | lentiCRISPR.*Pten*-sgRNA1 | This work |
| MLR-E1107 | *E. coli* | XL1 | lentiCRISPR.*Pten*-sgRNA2 | This work |
| MLR-E1108 | *E. coli* | XL1 | sgOpti.*Gsk3a*-sgRNA1 | This work |
| MLR-E1109 | *E. coli* | XL1 | sgOpti.*Gsk3a*-sgRNA2 | This work |
| MLR-E1110 | *E. coli* | XL1 | sgOpti.*Gsk3a*-sgRNA3 | This work |
| MLR-E1111 | *E. coli* | XL1 | sgOpti.*Gsk3b*-sgRNA1 | This work |
| MLR-E1112 | *E. coli* | XL1 | sgOpti.*Gsk3b*-sgRNA2 | This work |
| MLR-E1113 | *E. coli* | XL1 | sgOpti.*Gsk3b*-sgRNA3 | This work |
| MLR-E1114 | *E. coli* | XL1 | lentiCRISPR.*Usp22*-sgRNA1 | This work |
| MLR-E1115 | *E. coli* | XL1 | lentiCRISPR.*Usp22*-sgRNA2 | This work |
| MLR-E1116 | *E. coli* | XL1 | lentiCRISPR.*Taf13*-sgRNA1 | This work |
| MLR-E1117 | *E. coli* | XL1 | lentiCRISPR.*Taf13*-sgRNA2 | This work |
| MLR-E1118 | *E. coli* | XL1 | lentiCRISPR.*Nelfcd*-sgRNA1 | This work |
| MLR-E1119 | *E. coli* | XL1 | lentiCRISPR.*Nelfcd*-sgRNA2 | This work |
| MLR-E1120 | *E. coli* | XL1 | pLV.*Pten*-CR | This work |
| MLR-E1121 | *E. coli* | XL1 | pLV.*Pten*-CR-G129E | This work |
| MLR-E1122 | *E. coli* | XL1 | pLV.*Pten*-CR-G129R | This work |
| MLR-L0001 | *L. monocytogenes* | 10403S | serovar 1/2a | 1, 2 |
| MLR-L1123 | *L. monocytogenes* | 10403S | Δ*actA*, phage-cured | This work |
| MLR-L1124 | *L. monocytogenes* | 10403S | Δ*actA* pPL1.pHyper-GFP pPL2t.pHyper-RFP | This work |
| MLR-L1125 | *L. monocytogenes* | 10403S | Δ*actA* pPL1.pHyper-GFP pPL2t.p*actA*-RFP (“GFP-*Lm*”) | This work |
| MLR-L0551 | *L. monocytogenes* | 10403S | pPL2.pHyper-mCherry | 3 |
| MLR-L1068 | *L. monocytogenes* | 10403S | Δ*flaA* | 4 |
| MLR-L0022 | *L. monocytogenes* | 10403S | Δ*hly* | 5 |
| MLR-L0216 | *L. monocytogenes* | EGD-e | serovar 1/2a | 1 |
| MLR-L1126 | *L. monocytogenes* | HER1034 | serovar 1/2c | 6 |
| MLR-L1127 | *L. monocytogenes* | HER1082 | serovar 4b | 6 |
| MLR-L1128 | *L. monocytogenes* | HER1083 | serovar 4e, ATCC 19118 | 6 |
| MLR-L1129 | *L. monocytogenes* | HER1247 | serovar 1/2a | 6 |
| MLR-L1130 | *L. monocytogenes* | HER1537 | serovar 1/2a | 6 |
| MLR-L1131 | *L. monocytogenes* | Li2 | serovar 4b, ATCC 19115 | 6 |
| MLR-L0516 | *L. ivanovii* |  |  | 6 |
| MLR-L0432 | *L. innocua* | CLIP11262 |  | 7 |
| MLR-L1132 | *L. seeligeri* | SLCC3954 |  | 6, 8 |
| MLR-L1133 | *L. riparia* |  |  | 6 |
| MLR-L1134 | *L. newyorkensis* | FSL M6-635 |  | 6 |
| MLR-B0704 | *B. subtilis* | MB4 |  | 9 |

**SUPPORTING INFORMATION REFERENCES**

1. Bécavin, C., Bouchier, C., Lechat, P., Archambaud, C., Creno, S., Gouin, E., Wu, Z., Kühbacher, A., Brisse, S., Pucciarelli, M.G., et al. (2014). Comparison of Widely Used Listeria monocytogenes Strains EGD, 10403S, and EGD-e Highlights Genomic Differences Underlying Variations in Pathogenicity. mBio *5*, e00969-14. 10.1128/mBio.00969-14.
2. Bishop, D.K., and Hinrichs, D.J. (1987). Adoptive transfer of immunity to Listeria monocytogenes. The influence of in vitro stimulation on lymphocyte subset requirements. J Immunol *139*, 2005–2009.
3. Vincent, W.J.B., Freisinger, C.M., Lam, P., Huttenlocher, A., and Sauer, J.-D. (2016). Macrophages mediate flagellin induced inflammasome activation and host defense in zebrafish. Cell Microbiol *18*, 591–604. 10.1111/cmi.12536.
4. Cesinger, M.R., Daramola, O.I., Kwiatkowski, L.M., and Reniere, M.L. (2022). The Transcriptional Regulator SpxA1 Influences the Morphology and Virulence of Listeria monocytogenes. Infect Immun *90*, e00211-22. 10.1128/iai.00211-22.
5. Jones, S., and Portnoy, D.A. (1994). Characterization of Listeria monocytogenes pathogenesis in a strain expressing perfringolysin O in place of listeriolysin O. Infect Immun *62*, 5608–5613. 10.1128/iai.62.12.5608-5613.1994.
6. Kind gift from Meeske AJ, University of Washington.
7. Glaser, P., Frangeul, L., Buchrieser, C., Rusniok, C., Amend, A., Baquero, F., Berche, P., Bloecker, H., Brandt, P., Chakraborty, T., et al. (2001). Comparative Genomics of Listeria Species. Science *294*, 849–852. 10.1126/science.1063447.
8. Meeske, A.J., Jia, N., Cassel, A.K., Kozlova, A., Liao, J., Wiedmann, M., Patel, D.J., and Marraffini, L.A. (2020). A phage-encoded anti-CRISPR enables complete evasion of type VI-A CRISPR-Cas immunity. Science *369*, 54–59. 10.1126/science.abb6151.
9. Portnoy, D.A., Tweten, R.K., Kehoe, M., and Bielecki, J. (1992). Capacity of listeriolysin O, streptolysin O, and perfringolysin O to mediate growth of Bacillus subtilis within mammalian cells. Infect Immun *60*, 2710–2717. 10.1128/iai.60.7.2710-2717.1992.
