# Supplementary material for: A genome-wide screen in macrophages identifies PTEN as required for myeloid restriction of Listeria monocytogenes infection
Source: PLoS Pathog. 2023 May 22;19(5):e1011058. doi: 10.1371/journal.ppat.1011058 (PMC10237667; doi:10.1371/journal.ppat.1011058)
Supplement: S1 Fig — (A) Uptake of Lm by various cell types. Cells were infected with Lm 10403S at MOI = 1 (iBMM, BMDM, and THP-1), MOI = 10 (Caco-2), or MOI = 50 (TIB73). For iBMM, BMDM, and THP-1, gentamicin was added 30 minutes post-infection and intracellular CFU were quantified at 1 hour post-infection. For Caco-2 and TIB73, gentamicin was added 1 hour post-infection and intracellular CFU were quantified 90 minutes post-infection. (B) Uptake of various bacterial species by iBMMs. iBMMs were infected as in (A). All data are presented as the percentage of the initial inoculum that was internalized. Data are means and SEM of at least three biological replicates (represented by individual data points). (DOCX) [file ppat.1011058.s004.docx]

**
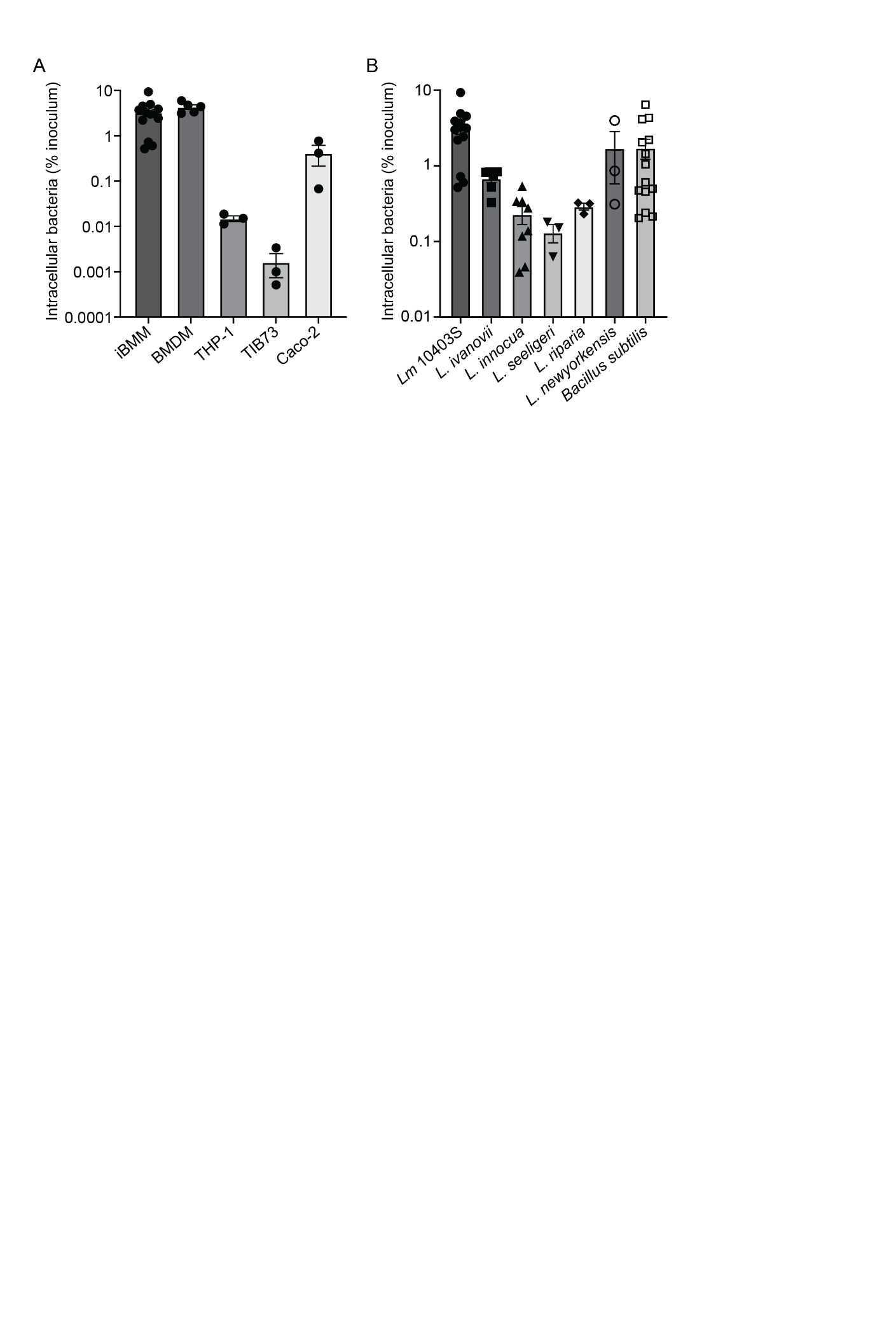
**

**S1 Fig. Raw data from gentamicin protection assays performed in this study.** (A) Uptake of *Lm* by various cell types. Cells were infected with *Lm* 10403S at MOI=1 (iBMM, BMDM, and THP-1), MOI=10 (Caco-2), or MOI=50 (TIB73). For iBMM, BMDM, and THP-1, gentamicin was added 30 minutes post-infection and intracellular CFU were quantified at 1 hour post-infection. For Caco-2 and TIB73, gentamicin was added 1 hour post-infection and intracellular CFU were quantified 90 minutes post-infection. (B) Uptake of various bacterial species by iBMMs. iBMMs were infected as in (**A**). All data are presented as the percentage of the initial inoculum that was internalized. Data are means and SEM of at least three biological replicates (represented by individual data points).
