# Supplementary material for: A genome-wide screen in macrophages identifies PTEN as required for myeloid restriction of Listeria monocytogenes infection
Source: PLoS Pathog. 2023 May 22;19(5):e1011058. doi: 10.1371/journal.ppat.1011058 (PMC10237667; doi:10.1371/journal.ppat.1011058)
Supplement: S2 Fig — (A) Gentamicin protection assay measuring bacterial uptake by iBMMs expressing Cas9. iBMMs were infected at MOI = 1 for 30 minutes and CFU were quantified 1 hour post-infection. Data are normalized to uptake of Lm. (B) Optimization of infection efficiency. iBMMs were infected with GFP-Lm for the indicated MOI and time, and GFP+ cells were quantified by flow cytometry 6 hours post-infection. (C) Hits with p<0.01 (n = 236) were analyzed by Metascape. The number of genes belonging to each biological process is depicted for the top 20 enriched pathways. Colors indicate pathways with similar functions. Data in (A-B) are means and SEM of three biological replicates. ***p<0.001 as determined by unpaired t tests. (DOCX) [file ppat.1011058.s005.docx]

**
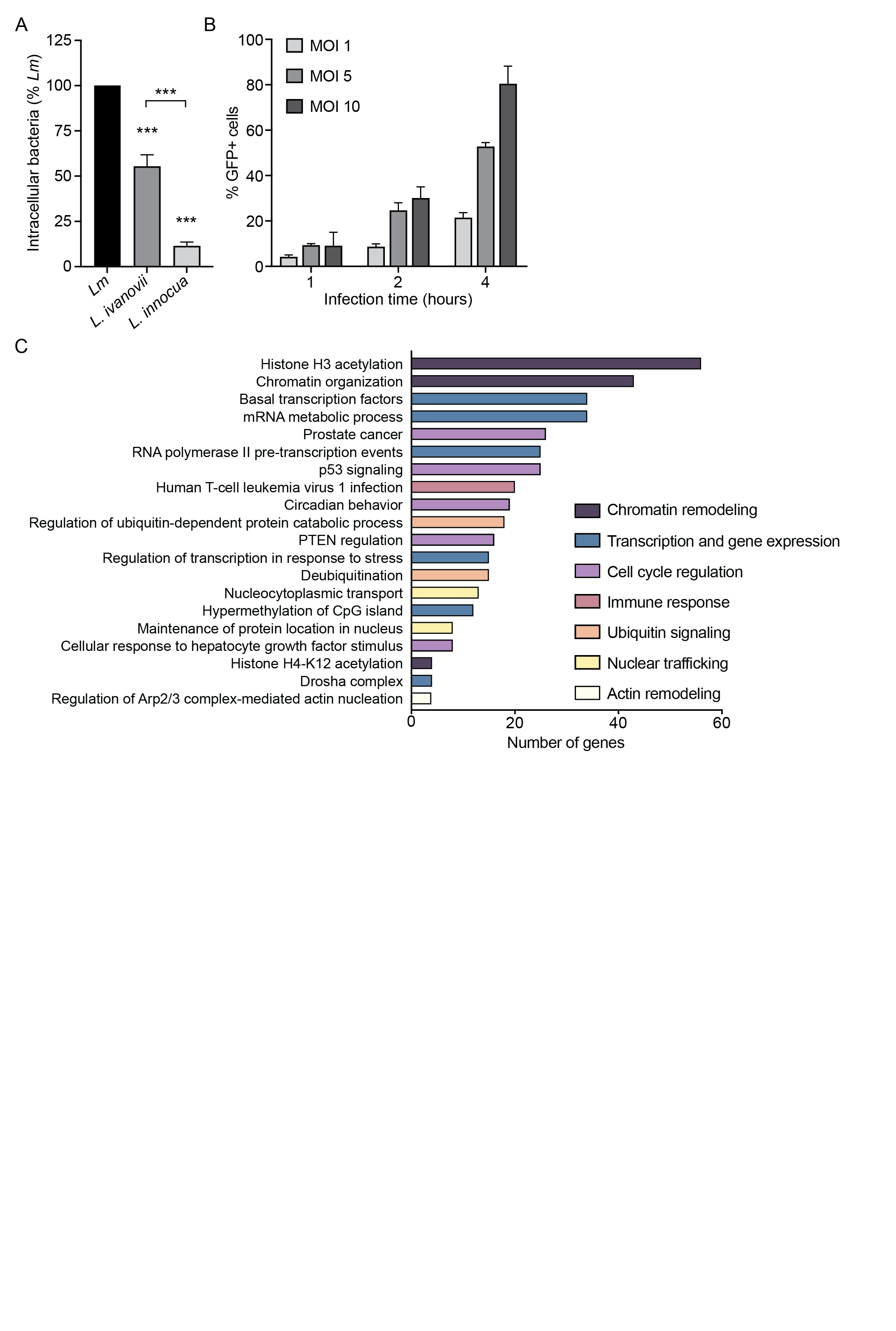
**

**S2 Fig. CRISPR screen optimization and Metascape analysis.** (A) Gentamicin protection assay measuring bacterial uptake by iBMMs expressing Cas9. iBMMs were infected at MOI=1 for 30 minutes and CFU were quantified 1 hour post-infection. Data are normalized to uptake of *Lm*. (B) Optimization of infection efficiency. iBMMs were infected with GFP-*Lm* for the indicated MOI and time, and GFP^+^ cells were quantified by flow cytometry 6 hours post-infection. (C) Hits with *p*<0.01 (n=236) were analyzed by Metascape. The number of genes belonging to each biological process is depicted for the top 20 enriched pathways. Colors indicate pathways with similar functions. Data in (A-B) are means and SEM of three biological replicates. ****p*<0.001 as determined by unpaired *t* tests.
