# Supplementary material for: A genome-wide screen in macrophages identifies PTEN as required for myeloid restriction of Listeria monocytogenes infection
Source: PLoS Pathog. 2023 May 22;19(5):e1011058. doi: 10.1371/journal.ppat.1011058 (PMC10237667; doi:10.1371/journal.ppat.1011058)
Supplement: S3 Fig — Gentamicin protection assay measuring uptake of Lm strains, L. ivanovii, and L. innocua by iBMMs. iBMMs were infected at MOI = 1 for 30 minutes and CFU were quantified 1 hour post-infection. The initial inoculum of each strain was enumerated and the percentage of internalized bacteria was calculated. Data are means and SEM of at least two biological replicates. (DOCX) [file ppat.1011058.s006.docx]

**
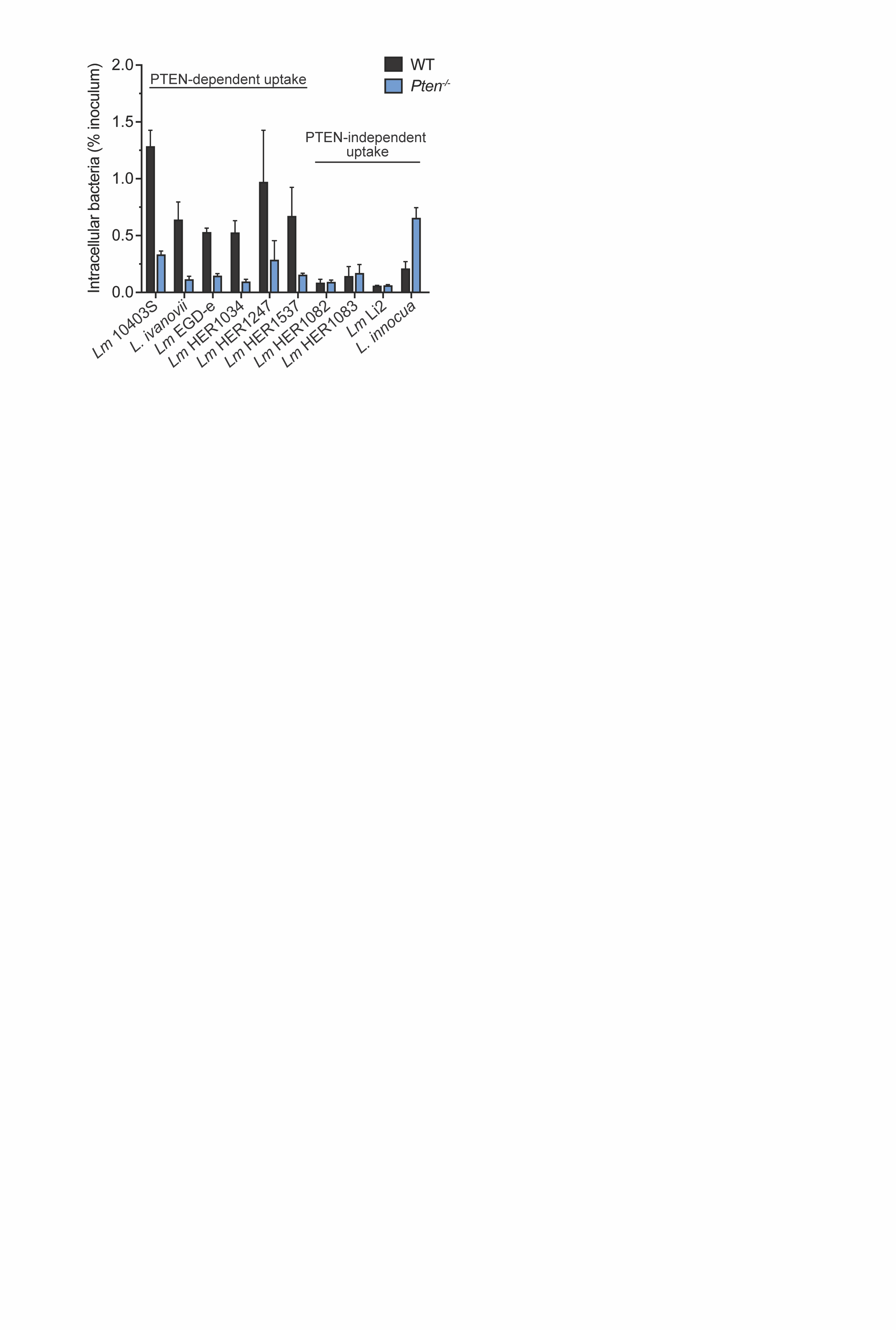
**

**S3 Fig.** **Enhanced phagocytosis of *Lm* is PTEN-dependent.** Gentamicin protection assay measuring uptake of *Lm* strains, *L. ivanovii*, and *L. innocua* by iBMMs. iBMMs were infected at MOI=1 for 30 minutes and CFU were quantified 1 hour post-infection. The initial inoculum of each strain was enumerated and the percentage of internalized bacteria was calculated. Data are means and SEM of at least two biological replicates.
