# Supplementary material for: A genome-wide screen in macrophages identifies PTEN as required for myeloid restriction of Listeria monocytogenes infection
Source: PLoS Pathog. 2023 May 22;19(5):e1011058. doi: 10.1371/journal.ppat.1011058 (PMC10237667; doi:10.1371/journal.ppat.1011058)
Supplement: S4 Fig — iBMMs were infected, immunostained, and quantified as in Fig 3C–3E. Timepoints were taken at 5, 15, 30, and 60 minutes post-infection. (A) Adherence and (B) internalization of Lm by iBMMs during the 1 hour time course were quantified. All data are means and SEM of three biological replicates. (DOCX) [file ppat.1011058.s007.docx]

**
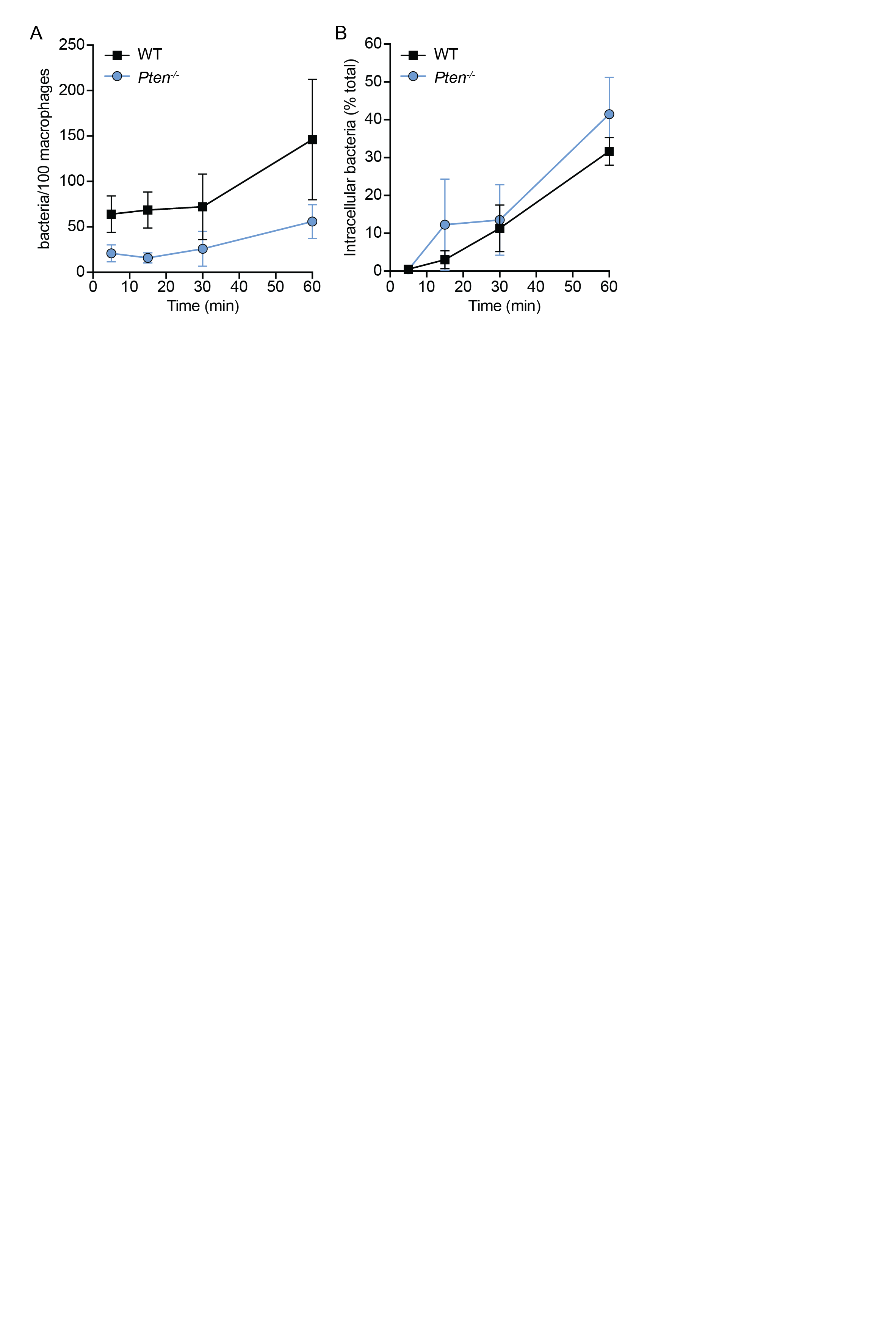
**

**S4 Fig. Dynamics of *Pten*^‒/‒^ iBMM infection.** iBMMs were infected, immunostained, and quantified as in Figure 3C-E. Timepoints were taken at 5, 15, 30, and 60 minutes post-infection. (A) Adherence and (B) internalization of *Lm* by iBMMs during the 1 hour time course were quantified. All data are means and SEM of three biological replicates.
