# Supplementary material for: A genome-wide screen in macrophages identifies PTEN as required for myeloid restriction of Listeria monocytogenes infection
Source: PLoS Pathog. 2023 May 22;19(5):e1011058. doi: 10.1371/journal.ppat.1011058 (PMC10237667; doi:10.1371/journal.ppat.1011058)
Supplement: S5 Fig — (A) Immunoblot analysis of TLR2 in WT and Tlr2–/– BMDMs. Na+/K+ ATPase was used as a loading control for membrane proteins. (B) Gentamicin protection assay measuring uptake of wildtype (wt) Lm by WT and Tlr2–/– BMDMs. Cells were infected at MOI = 1 for 30 minutes and CFU were quantified 1 hour post-infection. Data are normalized to WT BMDMs. (C) Gentamicin protection assay measuring uptake of Lm ΔflaA by WT and Tlr2–/– BMDMs. Data are normalized to WT BMDMs. (D) Gentamicin protection assay measuring uptake of Lm ΔflaA by WT and Tlr2–/– BMDMs in the presence of 5 μM bpV(pic) or 100 nM wortmannin. Data are normalized to vehicle-treated cells. (E) Immunoblot of phosphorylated Akt (Ser473) in WT and Tlr2–/– BMDMs mock-infected or infected with Lm wt or ΔflaA strains. BMDMs were infected with MOI = 100 and lysed 15 minutes post-infection. Total Akt and β-actin were used as loading controls. (F) Gentamicin protection assay measuring uptake of Lm 10403S, Lm Li2, or L. innocua by WT and Tlr2–/– BMDMs. Data are normalized to WT BMDMs for each strain. All data are means and SEM of at least three biological replicates except (F) which consists of two biological replicates. **p<0.01, ***p<0.001, as determined by unpaired t tests. (DOCX) [file ppat.1011058.s008.docx]

**
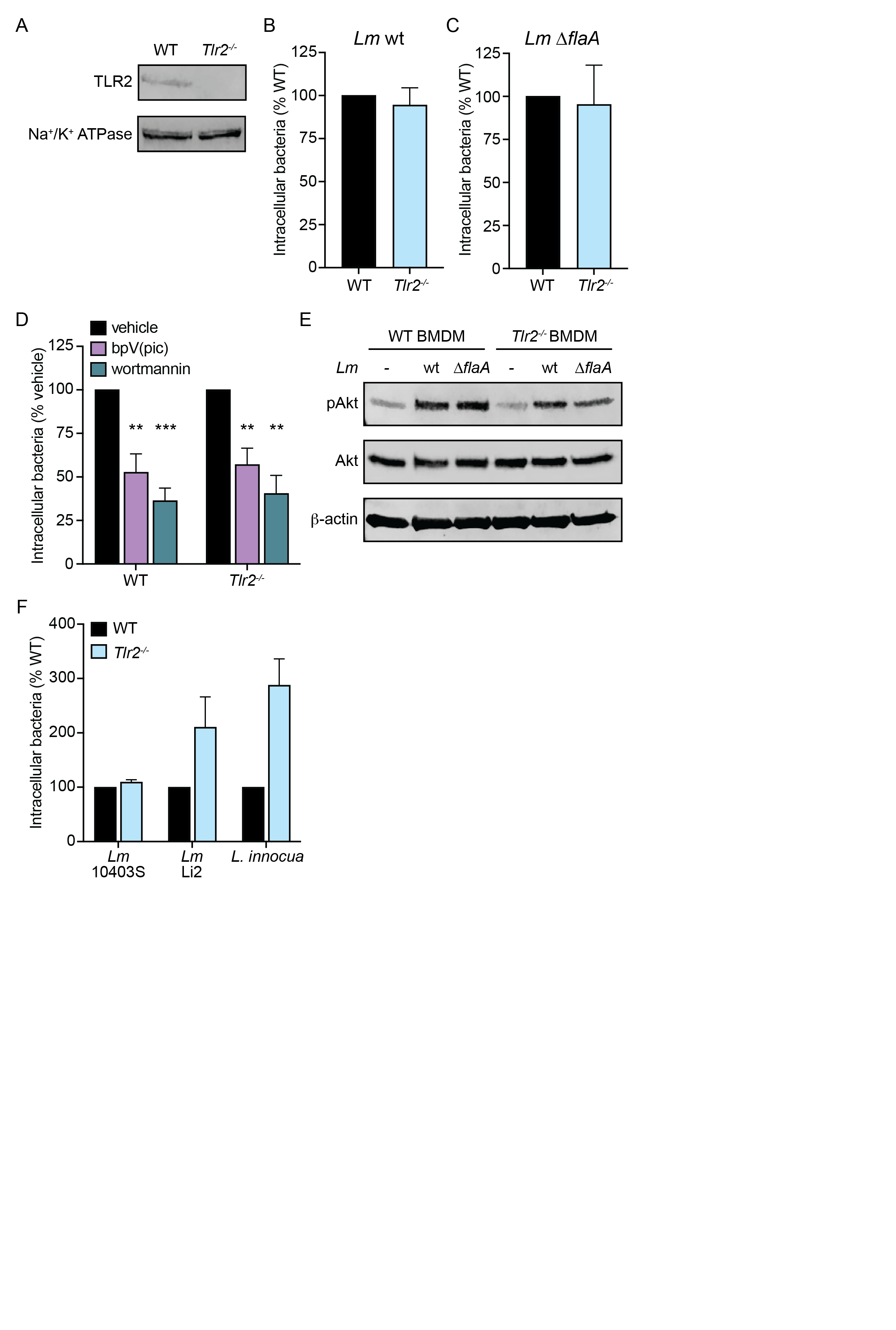
**

**S5 Fig. Uptake of *Listeria* by BMDMs is TLR-independent.** (A) Immunoblot analysis of TLR2 in WT and *Tlr2*^‒/‒^ BMDMs. Na^+^/K^+^ ATPase was used as a loading control for membrane proteins. (B) Gentamicin protection assay measuring uptake of wildtype (wt) *Lm* by WT and *Tlr2*^‒/‒^ BMDMs. Cells were infected at MOI=1 for 30 minutes and CFU were quantified 1 hour post-infection. Data are normalized to WT BMDMs. (C) Gentamicin protection assay measuring uptake of *Lm* Δ*flaA* by WT and *Tlr2*^‒/‒^ BMDMs. Data are normalized to WT BMDMs. (D) Gentamicin protection assay measuring uptake of *Lm* Δ*flaA* by WT and *Tlr2*^‒/‒^ BMDMs in the presence of 5 µM bpV(pic) or 100 nM wortmannin. Data are normalized to vehicle-treated cells. (E) Immunoblot of phosphorylated Akt (Ser473) in WT and *Tlr2*^‒/‒^ BMDMs mock-infected or infected with *Lm* wt or Δ*flaA* strains. BMDMs were infected with MOI=100 and lysed 15 minutes post-infection. Total Akt and β-actin were used as loading controls. (F) Gentamicin protection assay measuring uptake of *Lm* 10403S, *Lm* Li2, or *L. innocua* by WT and *Tlr2*^‒/‒^ BMDMs. Data are normalized to WT BMDMs for each strain. All data are means and SEM of at least three biological replicates except (F) which consists of two biological replicates. ***p*<0.01, ****p*<0.001, as determined by unpaired *t* tests.
