# Supplementary material for: A genome-wide screen in macrophages identifies PTEN as required for myeloid restriction of Listeria monocytogenes infection
Source: PLoS Pathog. 2023 May 22;19(5):e1011058. doi: 10.1371/journal.ppat.1011058 (PMC10237667; doi:10.1371/journal.ppat.1011058)
Supplement: S6 Fig — Mice were orally infected with 108 Lm 10403S. Bacterial burdens in (A) ileal and (B) colonic intestinal fractions were enumerated 3 dpi (MUC = mucus layer, EC = epithelial cells, LP = lamina propria). Each data point represents a single mouse (ileum, n = 4 per genotype; colon, n = 7 per genotype). Data for colonic fractions represent two independent experiments. Solid lines indicate geometric means. Dashed lines indicate the l.o.d. (DOCX) [file ppat.1011058.s009.docx]

**
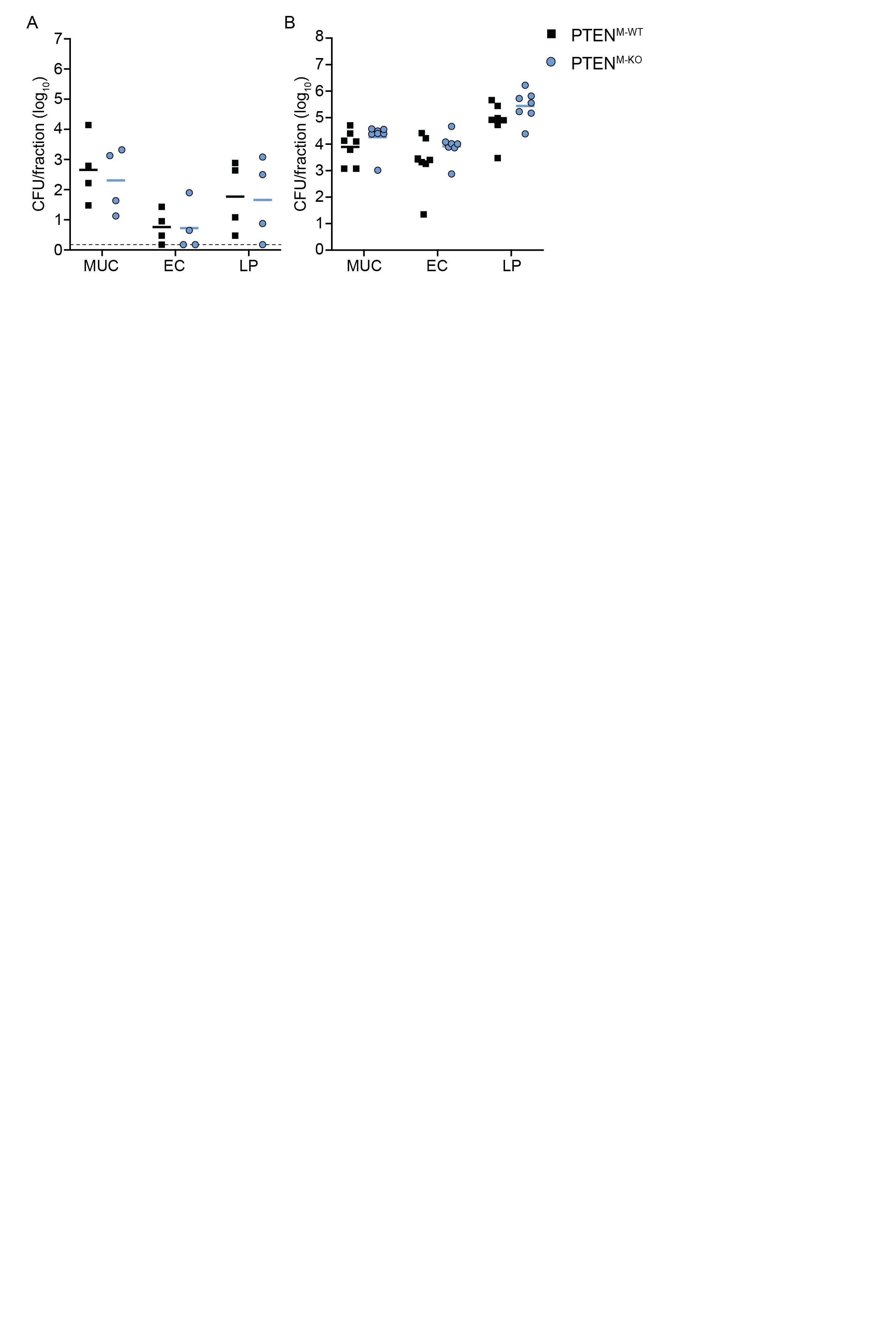
**

**S6 Fig. Intestinal fractions from 10403S-infected mice 3 dpi.** Mice were orally infected with 10^8^ *Lm* 10403S. Bacterial burdens in (A) ileal and (B) colonic intestinal fractions were enumerated 3 dpi (MUC = mucus layer, EC = epithelial cells, LP = lamina propria). Each data point represents a single mouse (ileum, n = 4 per genotype; colon, n = 7 per genotype). Data for colonic fractions represent two independent experiments. Solid lines indicate geometric means. Dashed lines indicate the l.o.d.
