# Supplementary material for: A genome-wide screen in macrophages identifies PTEN as required for myeloid restriction of Listeria monocytogenes infection
Source: PLoS Pathog. 2023 May 22;19(5):e1011058. doi: 10.1371/journal.ppat.1011058 (PMC10237667; doi:10.1371/journal.ppat.1011058)
Supplement: S7 Fig — (A-C) Mice were orally infected with 5 x 10^8^ Lm Li2 and CFU were enumerated from (A) feces, (B) ceca, and (C) livers 5 dpi. Each data point represents a single mouse from two independent experiments (n = 10 per genotype). (D-E) Mice were infected intravenously with 1.5 x 10^5^ Lm 10403S via retro-orbital injection and CFU were enumerated from (D) spleens and (E) livers 2 dpi. Each data point represents a single mouse (n = 5 per genotype). Solid lines indicate geometric means. Dashed line indicates the l.o.d. *p<0.05 as determined by unpaired t tests of natural log-transformed values. (DOCX) [file ppat.1011058.s010.docx]

**
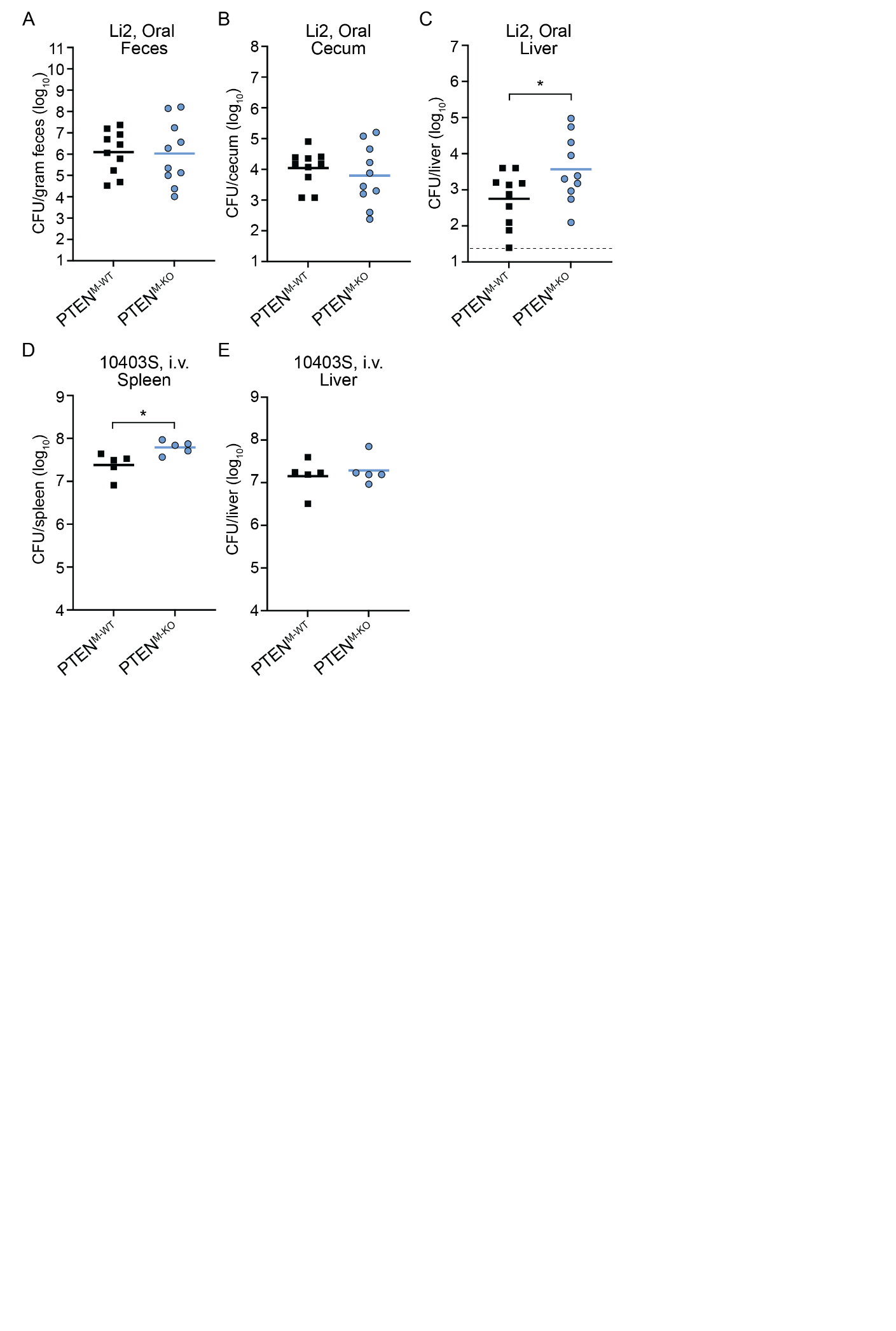
**

**S7 Fig.** **Myeloid PTEN restricts systemic *Lm* burden through regulation of phagocytosis.** (A-C) Mice were orally infected with 5 x 10^8^ *Lm* Li2 and CFU were enumerated from (A) feces, (B) ceca, and (C) livers 5 dpi. Each data point represents a single mouse from two independent experiments (n = 10 per genotype). (D-E) Mice were infected intravenously with 1.5 x 10^5^ *Lm* 10403S via retro-orbital injection and CFU were enumerated from (D) spleens and (E) livers 2 dpi. Each data point represents a single mouse (n = 5 per genotype). Solid lines indicate geometric means. Dashed line indicates the l.o.d. **p*<0.05 as determined by unpaired *t* tests of natural log-transformed values.
